# Impacts of Endogenous Retroviruses on Tumorigenesis, Immunity, Stem Cells, and Research Safety

**DOI:** 10.3389/fonc.2014.00066

**Published:** 2014-03-31

**Authors:** Iyoko Katoh

**Affiliations:** ^1^Molecular Biology, Center for Medical Education and Sciences, University of Yamanashi, Yamanashi, Japan

**Keywords:** human endogenous retrovirus, long terminal repeat, retrotransposon, endogenous retrovirus, carcinogenesis, stem cells

The human genome contains a category of repeat sequences referred to as human endogenous retroviruses (HERVs), which occupies up to 8% of the nucleotide sequences. HERVs have mutated and/or fragmented provirus structures of once-infectious retroviruses, and have lost their ability to replicate or transpose. Nonetheless, proteins encoded by different types of HERVs still show biological activities in various modes, and most of the regulatory regions termed “long terminal repeats” (LTRs) preserve the function as a promoter–enhancer region. Release from epigenetic silencing and activation of transcription factors can cause “awakening” of HERVs and LTRs involved in tumorigenesis, self-defense, and tissue development. This research topic is a compilation of unique reviews on HERVs and mouse endogenous retroviruses (ERVs) and original articles indicating new insights into HERVs.

## HERV-K(HML-2) as a Fossil Virus and a Functional Element

Human endogenous retroviruses-K(HML-2), the most recently integrated HERV family, preserve a nearly complete retrovirus structure, and has been an interesting subject in virology, genome biology, oncology, and pathology of autoimmune diseases. In the comprehensive Review Article (1), Hohn et al. outlined current knowledge on HERV-K(HML-2) including the topics of endogenization, genes-and-proteins, evolution, and tissue development. Moreover, the activation of HERV-K(HML-2) in diseases and the possibility of HERV-K-mediated oncogenesis were extensively discussed.

## Expression of HERV-K in Cancers

Agoni et al. contributed an Original Research Article (2) on the locus-specific analysis of HERV-K(HML-2) expression in prostate cancer cell lines. In addition to the previously known cancer types, melanoma, breast cancer, etc., prostate cancer was also found to express HERV-K and the LTR. Activation of specific HERV-K loci and an abundance of solo LTR-containing transcripts were described. As these authors mentioned, with deeper examination, this approach could possibly lead to determination of a clinical significance of HERV expression.

## HERV as the Target of Cancer Immunotherapy

Tumor cells express extra genes, such as HERVs, that are repressed in normal cells. If a HERV-derived protein is tumor-specific, it could be a useful marker in cancer diagnosis and therapy. As described by Cherkasova et al. (3), this hypothesis was proven by identification of the novel HERV-E-derived peptides of renal cell carcinomas as the target antigens in successful hematopoietic stem cell transplantation therapy. The authors discussed the mechanism of cancer-specific HERV expression as well as immunological responses to various types of HERV proteins.

## Great Confusion Caused by a Recombinant Mouse ERV in Cancer Research

In contrast to HERVs, ERVs of mouse laboratory strains currently remain active. Mice producing infectious viruses have provided interesting animal models for tumor biology in the past. Ironically, the nature of mouse ERVs has been recently “rediscovered” in human prostate cancer cells through contamination of a recombinant ERV termed xenotropic murine leukemia virus-related virus (XMRV). The Mini Review by Hempel et al. (4) covered the entire story, and raised alerts of the threat of XMRV-like virus spreading in laboratories, and for the virus-caused phenotypic changes of xenotransplanted cancer cells.

## Epigenetic Changes of Retroelements in Cancers

DNA hypomethylation is thought to be a major and common cause of the activation of retrotransposons in cancer development. Kreimer et al. (5) studied methylation status and expression of three retroelements, *LINE-1, HERV-K*, and *AluY*, in bladder cancer tissues and cell lines. They detected a considerable correlation between hypomethylation and transcription of *LINE-1*. Retroelements may undergo epigenetic control in different modes based on the tissues, subclasses, and chromosomal loci.

## HERVs as Non-Movable Transposons Associated with Human Diseases

Human endogenous retroviruses and HERV-derived LTRs, with mammalian apparent LTR-retrotransposons (MaLRs), compose the subclass of LTR-retrotransposons. Recent evidence indicated association of these elements with pathogenesis and a beneficial role in tumor therapy. Katoh and Kurata (6) summarized the influences of LTR-retrotransposons on human health and diseases, compared with presently active non-LTR-retrotransposons.

## Immune Regulation by *Mtv*, the Endogenous Form of Mouse Mammary Tumor Virus

Mouse mammary tumor virus and *Mtv* have attracted HERV scientists because of the structural and functional similarity between MMTV/*Mtv* and certain HERVs, and the presence of superantigen (Sag) in MMTV/*Mtv* and HERV-K18. Holt et al. (7) contributed a fresh Review focusing on *Mtv*, in which the impacts of this element on immune responses, infectious diseases, and tumor development were discussed in great detail.

## Mouse Endogenous Retrovirus MuERV-L/MERVL in Stem Cells

Studies on epigenetic regulation of ERVs in mouse stem cells are, although indirectly, meaningful for elucidation of the status of HERVs in development and carcinogenesis. Schoorlemmer et al. (8) explained silencing of MERVL, a distinct type of ERV expressed at the two-cell stage after fertilization, by histone modification with orchestration of multiple proteins. They offered an interesting hypothesis of MERVL LTR-mediated regulation of neighboring genes relevant to the stem cell potency.

All these articles which came from diverse backgrounds illustrated HERVs/ERVs from the basics to the frontlines, providing new perspectives. There was a great deal of discussion concerning the epigenetic control of HERVs/ERVs in embryonic development and tumorigenesis. HERVs and LTRs are emerging as genome inhabitants associated with human pathogenesis.
